# Adaptation of turnip mosaic potyvirus to a specific niche reduces its genetic and environmental robustness

**DOI:** 10.1093/ve/veaa041

**Published:** 2020-05-20

**Authors:** Anamarija Butković, Rubén González, Inés Cobo, Santiago F Elena

**Affiliations:** Instituto de Biología Integrativa de Sistemas (I2SysBio), CSIC-Universitat de València, Parc Cientific UV, Catedrático Agustín Escardino 9, Paterna, 46980 Valencia, Spain; Instituto de Biología Integrativa de Sistemas (I2SysBio), CSIC-Universitat de València, Parc Cientific UV, Catedrático Agustín Escardino 9, Paterna, 46980 Valencia, Spain; Instituto de Biología Integrativa de Sistemas (I2SysBio), CSIC-Universitat de València, Parc Cientific UV, Catedrático Agustín Escardino 9, Paterna, 46980 Valencia, Spain; Instituto de Biología Integrativa de Sistemas (I2SysBio), CSIC-Universitat de València, Parc Cientific UV, Catedrático Agustín Escardino 9, Paterna, 46980 Valencia, Spain; The Santa Fe Institute, 1399 Hyde Park Road, Santa Fe, NM 87501, USA

**Keywords:** experimental evolution, thermal fluctuations, mutagenesis, plant virus, plastogenetic congruence, robustness, virus evolution

## Abstract

Robustness is the preservation of the phenotype in the face of genetic and environmental perturbations. It has been argued that robustness must be an essential fitness component of RNA viruses owed to their small and compacted genomes, high mutation rates and living in ever-changing environmental conditions. Given that genetic robustness might hamper possible beneficial mutations, it has been suggested that genetic robustness can only evolve as a side-effect of the evolution of robustness mechanisms specific to cope with environmental perturbations, a theory known as plastogenetic congruence. However, empirical evidences from different viral systems are contradictory. To test how adaptation to a particular environment affects both environmental and genetic robustness, we have used two strains of turnip mosaic potyvirus (TuMV) that differ in their degree of adaptation to *Arabidopsis thaliana* at a permissive temperature. We show that the highly adapted strain is strongly sensitive to the effect of random mutations and to changes in temperature conditions. In contrast, the non-adapted strain shows more robustness against both the accumulation of random mutations and drastic changes in temperature conditions. Together, these results are consistent with the predictions of the plastogenetic congruence theory, suggesting that genetic and environmental robustnesses may be two sides of the same coin for TuMV.

## 1. Introduction

RNA viruses are very successful parasites that infect hosts across all biological kingdoms. This evolutionary success results from their evolvability, which in turn depends on the combination of three factors, namely high mutation rates, short generation times, and very large population sizes. However, these properties also come with costs. First, high mutation rates impose an upper limit to the length of the genome that can be maintained without increasing mutational load, which results in highly streamlined and compacted genomes (Elena and Sanjuán, 2005; Belshaw, Pybus, and Rambaut 2007). Second, most mutations have a deleterious fitness effect, with a large fraction of them being even lethal (reviewed in Sanjuán 2010), thus jeopardizing the survival of viral populations. How do RNA viruses maintain their functionality under such scenario of strong genomic stress? In the last 15 years or so, several studies have experimentally shown that such mutational pressure favors mechanisms that promote mutational robustness in RNA viruses (e.g. Montville et al. 2005; Codoñer et al. 2006; Sanjuán et al. 2007; Stern et al. 2014; Thyagarajan and Bloom 2014; Visher et al. 2016). Broadly speaking, genetic robustness refers to the constancy of the phenotype in the face of heritable perturbations (genetic or epigenetic; de Visser et al. 2003). However, the evolutionary origin and maintenance of genetic robustness still remains an unsolved question (de Visser et al. 2003; Elena et al. 2006; Elena 2012; Lauring, Frydman, and Andino 2013). Any mutation increasing genetic robustness will hardly rise in frequency because they have no other phenotypic effect than buffering the effect of other mutations (de Visser et al. 2003). This means that: 1, they will increase in frequency only at very high deleterious mutation rates because genotypes without these robustness-conferring mutations will simply suffer stronger mutational loads. 2, They will slow down the rate of adaptation by buffering the effect of other linked beneficial mutations. In conclusion, at low deleterious mutation rates (which may not be the case of RNA viruses), genetic robustness will not be easily selected. In theory, genotypes that produce more neutral mutations (i.e. they inhabit in neutral network within the genotypic landscape) could be directly selected (Wilke 2001; Wilke et al. 2001; Codoñer et al. 2006). However, plenty of mutation-accumulation studies done with different RNA viruses suggest that the fraction of neutral mutations should be relatively small compared with those having deleterious effects (Sanjuán 2010). Mutation accumulation in small populations may also select for genetic robustness (Krakauer and Plotkin 2002; Forster, Adami, and Wilke 2006; Elena et al. 2007), though a low population size would also reduce the effectiveness of selection (Forster, Adami, and Wilke 2006; Elena et al. 2007).

How to escape from this conundrum? In this context is where Ancel and Fontana (2000) postulated the plastogenetic congruence theory. Rapid environmental fluctuations and environmental unpredictability are quite common selective pressures and, therefore, any mutation conferring environmental robustness will necessarily be efficiently selected. Taken in a broad sense, environmental robustness refers to any kind of buffering against non-heritable perturbations (including both external stresses and developmental noise caused by fluctuations in the concentration of morphogens; de Visser et al. 2003). The plastogenetic congruence theory postulates that genetic robustness will arise as a correlated trait of strong selection for environmental robustness.

Viruses face strongly unpredictable environments during their life cycles: heterogeneity in susceptible host species, differences in cell types and even in the physiological stages of susceptible cells within a host species, the presence of antiviral immune and pharmacological responses, and other environmental factor, being temperature a well-known driver of virus adaptation (González, Butković, and Elena 2020). Experimental support to the plastogenetic congruence hypothesis in viruses was provided by Domingo-Calap, Pereira-Gómez, and Sanjuán (2010), who evolved populations of bacteriophage Qβ under periodic temperature pulses to select for thermotolerant viruses (i.e. environmentally robust) that in a series of subsequent experiments were shown to be also more genetically robust than control viruses.

In this study, we tested the plastogenetic congruence hypothesis using turnip mosaic virus (TuMV; genus *Potyvirus*, family *Potyviridae*) in its natural host the model plant *Arabidopsis thaliana* (L.) Heynh. Specifically, we have used two strains of TuMV that differ in their degree of adaptation to Arabidopsis. The first one was originally isolated from calla lily and was not well adapted to Arabidopsis, hereafter referred to as TuMV-AS. The second one, obtained after twelve passages of experimental evolution of TuMV-AS in the Arabidopsis ecotype Col-0, shows a high degree of adaptation; we will refer to this adapted strain as TuMV-DV. In our study, we have evaluated the mutational and environmental robustness (thermal stability) of both strains. We found that TuMV-DV was very fragile to the accumulation of random mutations and showed very little thermostability. In contrast, TuMV-AS was more robust both mutationally and environmentally. We discuss these results in the context of the plastogenetic congruence hypothesis and also in the context of how adaptation to one environment limits evolvability in alternative ones.

## 2. Methods

### 2.1. Viruses, plants, and inoculations

As a source of the inocula for all experiments described below, we used stocks of infectious saps from Arabidopsis Col-0 infected plants. Saps were obtained by grinding the corresponding infected tissues in a mortar with ten volumes of grinding buffer (50 mM KH_2_PO_4_ pH 7, 3 per cent polyethylene glycol 6000). In the case of TuMV-AS, an Arabidopsis-naïve virus, *Nicotiana benthamiana* Domin plants were inoculated with the plasmid p35STunos that contains a cDNA of TuMV isolate YC5 from calla lily (*Zantedeschia* sp.; GenBank accession AF530055.2) under the control of the cauliflower mosaic virus 35S promoter and the *nos* terminator (Chen et al. 2003). A large stock of viral particles was produced from these plants. In the case of TuMV-DV, the virus was obtained after twelve serial passages of experimental evolution in Arabidopsis Col-0 of the ancestral TuMV-AS isolate (González, Butković, and Elena 2019; Navarro et al. in prep.), thus representing the case of an Arabidopsis-adapted virus.

Arabidopsis plants were always inoculated when they reached growth stage 3.5 in the Boyes’ scale (Boyes 2001). Aliquots of 5 μl of 10 per cent Carborundum in grinding buffer were applied onto three different leaves, and inoculation was done mechanically by gentle rubbing with a glass stick.

Unless otherwise indicated, plants were maintained in a BSL-2 growing chamber at 16-h light:8-h dark cycles and temperature variation of 24°C day:20°C night. Plants that showed visible symptoms of infection were harvested 14 days post-inoculation (dpi).

### 2.2. Evaluation of mutational robustness

N_2_O mutagenesis was done as described in Willemsen et al. (2018). In short, ground-infected tissues were homogenized with DEPC-treated sterile water at 1:1 (w:v) ratio. Diluted saps were centrifuged 2 min at 12,000 rpm at 4°C and the supernatant was transferred into two different tubes. The first tube contained a control reaction consisting of equal volumes of water and 0.5 M sodium acetate (pH 5.4). The second tube contained the mutagenic reaction consisting in equal volumes of 2 M NaNO_3_ and 0.5 M sodium acetate (pH 5.4). These tubes were incubated at 26°C for 3 h. After incubation, 1/10th volume of 0.5 M phosphate buffer (pH 7) was added to the tubes to stop the mutagenic reactions.

Four groups of twelve plants were inoculated each with mutagenized and non-mutagenized versions of TuMV-AS and TuMV-DV. Inoculated plants were maintained in the standard growth conditions described in Section 2.1 during 21 dpi.

### 2.3. Evaluation to thermal robustness

All plants were maintained in the standard cultivation conditions described in Section 2.1 from germination until 1 week before inoculation. During this week, plants were acclimatized to the thermal conditions corresponding to each of the following four experimental condition (twenty-four plants each): 1, constant 24°C; 2, constant 30°C; 3, sequential changes between 15°C, 24°C, and 30°C every 24 h (median temperature across the entire experiment 24.0°C, IQR 13.5°C); and 4, random changes between 15°C, 24°C, and 30°C every 24 h (median temperature across the entire experiment 24.0°C, IQR 15.0°C). In all four setups, illumination conditions remained 16-h light and 8-h dark. After this acclimation week, plants were inoculated; 12 with TuMV-AS and 12 with TuMV-DV, and kept in the corresponding thermal regime during 21 dpi. Treatments (3) and (4) were designed to increase the amount of environmental noise to which the replicating TuMV population would be exposed. The possibility of adding an additional constant 15°C treatment was discarded after some preliminary experiments because infections progressed asymptomatic and with very low viral loads (data not shown).

### 2.4. Disease progression curves as a proxy to the degree of viral adaptation

All inoculated plants were observed daily for 21 dpi for the presence of symptoms and the number of symptomatic plants recorded. Disease progression curves were characterized by three parameters, the median time to the development of visible symptoms (*ST*_50_), the final frequency of infected plants, or infectivity, (*I*) and the area under the disease progress stairs (*AUDPS*; Simko and Piepho 2012). *AUDPS* represents the intensity at which symptoms appear in a population of inoculated plants, and in our case, it is bounded between zero (no plant shows symptoms 21 dpi) and twelve (all plants show symptoms at 1 dpi).

In the TuMV/Arabidopsis pathosystem, there is a one-to-one match between infection status and the development of symptoms (González, Butković, and Elena 2019; Corrêa et al. 2020); all infected plants develop obvious symptoms at the temperature conditions used in this experiment. Likewise, in this pathosystem the intensity of symptoms is significantly correlated with viral load (Corrêa et al. 2020). Symptoms started with leaf curling and vein clearing (∼5–6 dpi) that quickly developed to diverse grades of leaf chlorosis and/or necrosis (∼10–12 dpi). Plants also suffered a developmental arrest, with deformed new leaves, siliques abortion, and abnormal growth of the caulinar apex.

### 2.5. Statistical analyses

The disease progression curves were analyzed using Kaplan–Meier survival regression analyses as implemented in SPSS version 26 software (IBM, Armonk, NY). The significance of factor effects was evaluated using the log-rank Mantel-Cox test statistic that asymptotically follows a *χ*^2^ distribution.

Infection data for each treatment were organized in a 12 × 22 binary matrix, where rows represent individual plants and columns dpi. Infection status was coded as 1 if plants showed symptoms and 0 otherwise. *AUDPS* values were computed using the ‘agricolae’ R package version 1.3-2 (https://tarwi.lamolina.edu.pe/∼fmendiburu/). Confidence intervals (95% CIs) were estimated using a bootstrapping method consisting in sampling with replacement the matrix rows, thus preserving the temporal correlations across time points. A thousand pseudo-replicated matrices of equal dimensions to the original one were obtained per experimental condition, thus generating kernel distributions for *AUDPS*. The median *AUDPS*s and their corresponding 95 per cent CIs were estimated from these distributions. This algorithm was implemented in R version 3.6.1 in RStudio version 1.2.1335.

A measure of environmental robustness is the inverse of the environmental variance, $\sigma_{E}^{2}$, which results from external environmental perturbations (de Visser et al. 2003). Variance components in a one-way ANOVA model testing for differences among thermal environments were estimated by maximum likelihood techniques as implemented in SPSS version 26 software (IBM, Armonk, NY). Net differences among thermal environments correspond to $\sigma_{E}^{2}$, whereas differences among replicates within a given environment correspond to random noise.

## 3. Results

The three variables measured, *AUDPS*, *I*, and *ST*_50_, were strongly correlated, as indicated by partial correlation analyses controlling for the viral isolate: *AUDPS* and *I* were positively correlated (*r_p_* = 0.9444, 7 df, *P *=* *0.0001), *AUDPS* and *ST*_50_ were negatively correlated (*r_p_* = −0.9965, 7 df, *P *<* *0.0001) and *I* and *ST*_50_ were negatively correlated too (*r_p_* = −0.9478, 7 df, *P *=* *0.0001). Fast appearance of symptoms (smaller *ST*_50_) and a large number of infected plants (larger *I*) are thus reflected in larger *AUDPS* values, thus confirming *AUDPS* provides a good proxy to the degree of adaptation of a particular viral genotype to its host and environmental conditions. Therefore, for simplicity, in the following sections, we will only report the results for the analyses done with *AUDPS*.

### 3.1. Adaptation of TuMV to Arabidopsis and standard thermal conditions results in a reduction in genetic robustness

**Figure 1.**
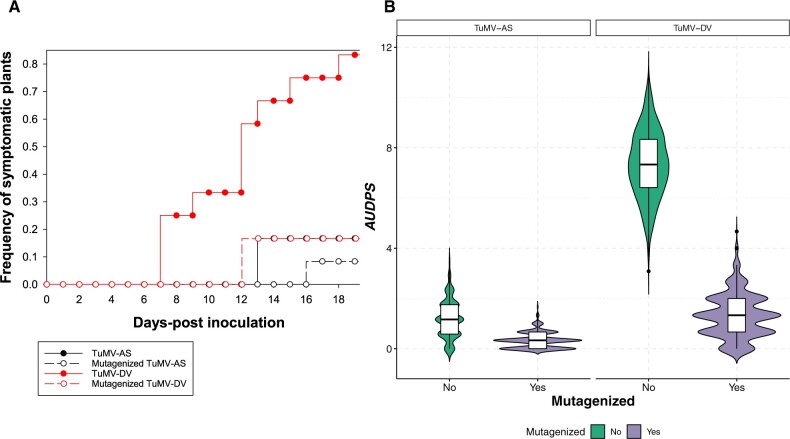
Evaluation of genetic robustness for the Arabidopsis-naïve (TuMV-AS) and Arabidopsis-adapted (TuMV-DV) viruses. (A) Disease progression curves for viruses submitted to N_2_O-induced mutagenesis (open symbols and dashed lines) and their corresponding non-mutagenized controls (solid symbols and lines). (B) Estimates of *AUDPS* for each experimental condition. The kernel distributions estimated using the bootstrap algorithm are over imposed to the box diagrams.

First, we evaluated the degree of adaptation to Arabidopsis Col-0 in standard growing conditions of both viruses. Figure 1A shows the disease progression curves for the naïve TuMV-AS (solid black symbols and lines) and the Arabidopsis-adapted TuMV-DV (solid red symbols and lines) viruses. Very significant differences exist between both viruses in the disease progression (*χ*^2^ = 11.9775, 1 df, *P *=* *0.0005). Consistently, the median *AUDPS* for TuMV-AS was 1.1667 ± 0.0463 (±95% CI), while it was 7.3333 ± 0.0785 for TuMV-DV (Fig. 1B, green distributions; i.e. 6.29-fold better adapted).

After confirming the higher degree of adaptation of TuMV-DV to Arabidopsis Col-0 in the standard growing conditions, we sought to evaluate the degree of genetic robustness of each one virus. Figure 1A shows the disease progression curves for the mutagenized viruses (open black symbols and dashed lines for TuMV-AS and open red symbols and dashed lines for TuMV-DV). Here we have compared mutagenized and non-mutagenized viruses. In the case of the non-adapted TuMV-AS isolate, the N_2_O mutagenic treatment had no significant effect in the disease progression curve (*χ*^2^ = 0.4097, 1 df, *P *=* *0.5221). The estimated median *AUDPS* for the mutagenized TuMV-AS was 0.3333 ± 0.0196 (Fig. 1B, purple distributions). In sharp contrast, in the case of the Arabidopsis-adapted TuMV-DV isolate, random mutagenesis had a strong negative effect on the progression curves (*χ*^2^ = 10.9902, 1 df, *P *=* *0.0009), with the median *AUDPS* estimated for the mutagenized TuMV-DV being 1.3333 ±0.0529 (Fig. 1B, purple distributions), which means a reduction of 81.82% in disease progression efficiency.

The conclusion from this first experiment is that adaptation to Arabidopsis Col-0 was concomitant with a decrease in genetic robustness. This observation is consistent with the notion of TuMV-DV inhabits a high but narrow fitness peak while TuMV-AS occupied a flatter and more neutral region of the fitness landscape.

### 3.2. TuMV-AS and TuMV-DV differ in environmental robustness

**Figure 2.**
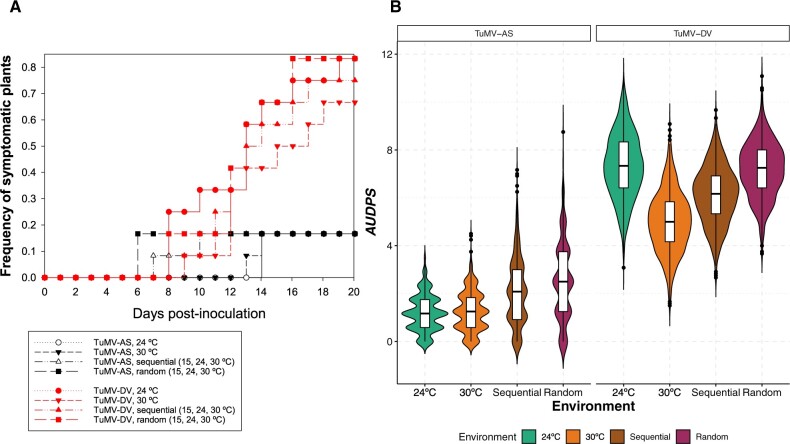
Evaluation of environmental robustness for the Arabidopsis-naïve (TuMV-AS) and Arabidopsis-adapted (TuMV-DV) viruses. (A) Disease progression curves for viruses growing under the four different thermal regimes (black symbols and lines for TuMV-AS and red symbols and lines for TuMV-DV). (B) Estimates of *AUDPS* for each experimental condition. The kernel distributions estimated using the bootstrap algorithm are over imposed to the box diagrams.

Next, we sought to evaluate the environmental robustness of both viral isolates. First, we found that no significant differences exist among the disease progression curves observed for TuMV-AS across the four thermal environments (Fig. 2a, black lines and symbols: *χ*^2^ = 0.3779, 1 df, *P *=* *0.5387). Again, in sharp contrast with this result, highly significant differences have been observed for the TuMV-DV across the four thermal environments (Fig. 2A, red lines and symbols: *χ*^2^ = 8.7213, 1 df, *P *=* *0.0031).

Interestingly, the variance component explained by differences among the four thermal environments was $\sigma_{E}^{2}$ = 0.7636 ±0.0171 (±1 SEM; maximum likelihood estimator of variance components in a one-way ANOVA) for TuMV-AS and $\sigma_{E}^{2}$ = 1.3132 ±0.0294 for TuMV-DV (Fig. 2B); that is 71.97% more variance among thermal environments in the latter.

These two results together suggest that TuMV-AS generates more consistent disease progression curves across the four thermal environments than the Arabidopsis-adapted TuMV-DV, which shows more variable responses across thermal environments. In other words, TuMV-AS is more environmentally robust (lower $\sigma_{E}^{2}$) than TuMV-DV.

## 4. Discussion

### 4.1. The tradeoff between robustness and evolvability in RNA viruses

The robustness of biological systems has several important implications. At the one side, it directly affects the probability of survival of organisms in the face of endogenous (i.e. genetic and epigenetic mutations) and exogenous (i.e. environmental uncertainties or developmental noise) perturbations (de Visser et al. 2003; Wagner 2005; Bloom et al. 2006; Ciliberti, Martin, and Wagner 2007; Wagner 2008a), thus being a beneficial fitness trait. At the other side, however, robustness and evolvability represent the two faces of the same coin; genetic robustness may slow down the rate of adaptation by masking the effect of beneficial mutations as much as it buffers the effect of deleterious ones. Evidences showing this negative association between genetic robustness and evolvability have been somehow contradictory. Experimental results with digital organisms (Elena and Sanjuán 2008) and vesicular stomatitis virus (VSV; Cuevas, Moya, and Sanjuán 2009) have shown a negative association between short-term adaptability and genetic robustness. In contrast, experiments with bacteriophages have shown the opposite trend: genetic robustness promotes the evolution of thermal stability (McBride, Ogbunugafor, and Turner 2008). Aligning with the bacteriophage results, Turner et al. (2010) have shown that environmentally robust (i.e. generalists) populations of VSV were also more evolvable than highly specialized populations.

How to reconciliate all these apparently contradictory results? First, it has been suggested that genetic robustness can facilitate or jeopardize adaptation depending on population size, mutation rate, and the topography of the underlying fitness landscape (Krakauer and Plotkin 2002; Draghi et al. 2010). Second, the relationship between robustness and evolvability may be time-dependent. At the short-term genetic robustness will buffer the effect of potentially beneficial mutations, thus hampering adaptation. However, at the long-term genetic robustness will bolster evolvability by allowing populations to drift within neutral networks until reaching distant parts and switching to different neutral networks (Elena and Sanjuán 2008; Wagner 2008b). The epochal evolution of influenza A virus H3N2, alternating periods of phenotypic stasis punctuated by sudden changes in antigenic phenotypes (Koelle et al. 2006) fits well within this model of time-dependent effects of robustness: at the onset of an epochal evolution cycle, a H3N2 population is distributed over the neutral network of an antigenic cluster. Neutral mutations accumulate, allowing the virus to explore distant regions of the network. Later on, genotypes reach the edge of the network and create individuals that belong to a new antigenic cluster (Koelle et al. 2006; van Nimwegen 2006).

### 4.2. The evolutionary origin of genetic robustness in RNA viruses

Still, the question of how genetic robustness evolves needs to be answered. An interesting proposal brought forward by Ancel and Fontana (2000) was the so-called plastogenetic congruence hypothesis. Under this hypothesis, genetic robustness evolves as a consequence of strong selection for mechanisms reducing the impact of environmental perturbations, that is, environmental robustness. Environmental perturbations along the life cycle of viruses occur constantly, thus imposing a strong selective advantage to any mechanism that may buffer them. How much evidence exists supporting the plastogenetic congruence hypothesis in the case of viruses? Domingo-Calap, Pereira-Gómez, and Sanjuán (2010) directly tested the hypothesis by evolving bacteriophage Qβ under fluctuating temperatures to select for thermotolerant viruses. Then, these viruses were submitted to accumulation of random mutations in the same way we have used in this study. Their results provided support to the hypothesis, as the more thermotolerant viruses were also more robust against the deleterious effect of accumulated mutations. Here, we have also found an association between genetic and environmental robustness for two TuMV strains that differed in their degree of adaption to Arabidopsis: the ancestral TuMV-AS shows more environmental robustness than its Arabidopsis-adapted descendant TuMV-DV, echoing the observed differences in mutational robustness. Together these studies provide evidences supporting the link between genetic and environmental robustnesses, though a mechanistic explanation for such link is still missing.

### 4.3. Virus specialization limits evolvability

Here, we have observed that TuMV adaptation to a particular Arabidopsis genotype (Col-0) and temperature conditions may be hampering its capacity to quickly respond to future changes in temperature. This observation mirrors the results of Turner et al. (2010) mentioned in Section 4.1, in which specialist populations of VSV where less evolvable to new cell types than generalist populations. A similar observation was done by Buckling, Wills, and Colegrave (2003) when exploring the evolvability of *Pseudomonas fluorescens* into different ecological niches. These authors argued that by climbing an adaptive peak, a population reduces standing genetic variability that would be beneficial in alternative environments, thus specializing into this particular niche. In contrast, a generalist population would exist outside of any particular fitness peak, gaining access to all of them (Buckling, Wills, and Colegrave 2003; Elena and Sanjuán 2003). In this sense, by specializing to complete its infection cycle at 24°C day temperature, TuMV-DV has limited its own evolvability.

### 4.4. Concluding remarks

In conclusion, we have shown results suggesting an association between environmental and genetic robustness in a natural pathosystem constituted by a plant virus and its natural multicellular eukaryotic host. This observation represents one small step forward in our understanding of the evolution of genetic robustness and adds generality to previous *in vitro* studies with bacteriophages and VSV. However, we still need to dig into the molecular and physiological mechanisms of such association between genetic and environmental robustness and the degree of adaptation to the host and growth conditions. An intriguing question is how much of the observed pattern is due to genomic changes in the virus versus the virus taking advantage from the host responses to thermal stress. For instance, it is well known that viruses take advantage of heat shock proteins (Elena et al. 2006; Geller et al. 2007) from the host, and thus an overexpression of such proteins by plant cells upon thermal stress may indirectly benefit the virus replication. This and similar questions will be explored in future works.
